# Association of cerebral spinal fluid copper imbalance in amyotrophic lateral sclerosis

**DOI:** 10.3389/fnagi.2022.970711

**Published:** 2022-11-17

**Authors:** Qiao Yi Chen, Peng Wu, Ting Wen, Xing Qin, Ronghua Zhang, Rui Jia, Jiaoting Jin, Fangfang Hu, Xiaoge Xie, Jingxia Dang

**Affiliations:** ^1^Department of Cell Biology and Genetics, School of Basic Medical Sciences, Xi’an Jiaotong University, Xi’an, China; ^2^Department of Neurology, The First Affiliated Hospital of Xi’an Jiaotong University, Xi’an, China

**Keywords:** ALS, copper, metal/metalloids, CSF, neurodegenerative

## Abstract

A plethora of environmental risk factors has been persistently implicated in the pathogenesis of amyotrophic lateral sclerosis (ALS), including metal/metalloids. This study aimed to examine potential associations between cerebral spinal fluid (CSF) metal/metalloids and ALS risks. CSF concentrations of copper (Cu), nickel (Ni), mercury (Hg), arsenic (As), manganese (Mn), and iron (Fe) in ALS (spinal- and bulbar-onset) patients and controls were measured using inductively coupled plasma mass spectrometry (ICP-MS). Results from this study revealed marked differences between control, spinal-onset, and bulbar-onset groups. We report that Cu levels were lower in the ALS and spinal-onset groups compared to the control group. Ni level were higher in the spinal-onset group compared to the control and bulbar-onset groups. In addition, associations between CSF metal/metalloid levels with disease severity, sex, and serum triglycerides were also examined to broach the potential relevance of neurotoxic metal/metalloids in ALS disease heterogeneity.

## Graphical Abstract

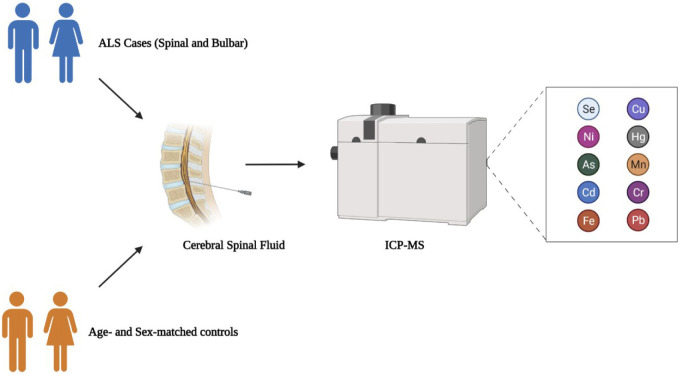

## Introduction

Amyotrophic lateral sclerosis (ALS), also referred to as “Lou Gehrig’s disease,” is a debilitating neurodegenerative disease with an incredibly intricate etiology. ALS manifestation is theorized as a stepwise process that involves both susceptible variants and environmental triggers (Al-Chalabi and Hardiman, 2013; Paez-Colasante et al., 2015; Cook and Petrucelli, 2019). The multifaceted pathogenic mechanisms coupled with genetic variability and complex environmental exposure may be important reasons for the complex biological heterogeneity of ALS, as well as why many preclinical ALS treatment trials fail in phase 3. Notably, a proportion of ALS patients (∼10%) show Mendelian inheritance while the majority (∼90%) of cases occur sporadically with no familial history. Moreover, at least 30 genes have been correlated with ALS.

Namely, C9orf72, TARDBP, SOD1, and FUS account for 70% of familial ALS cases in European populations (Chiò et al., 2014). However, unlike schizophrenia patients who show a number of common variants, ALS pathogenesis is primarily based on rare variants (van Rheenen et al., 2016). Large population-based analyses have suggested that certain risk variants have higher susceptibility for disease manifestation when coupled with environmental risk factors (Hardiman et al., 2017). Early case-control studies conducted in Guam and Japan suggested that exposure to cyanotoxins is correlated with high ALS susceptibility (Bradley et al., 2013). Other risk factors include military service, metals/metalloids, pesticides and insecticides, electromagnetic fields, physical activity, head injury, glutamate toxicity, and smoking (Talbott et al., 2016). At the molecular level, impairment in RNA metabolism, protein homeostasis, neuroinflammation and mitochondrial dysfunction have all been evidenced in ALS pathogenesis (Higgins et al., 2003; Wang et al., 2011; Brites and Vaz, 2014; Magrané et al., 2014; Sama et al., 2014; Conicella et al., 2016; Hardiman et al., 2017). It is important to note that dysregulations of the above cellular processes in ALS are likely to interact and culminate leading to disruptions in the broader mechanistic network, and that it is unlikely that any one of the dysregulated mechanisms is singularly responsible for ALS pathogenesis. The same should be considered when investigating the roles of environmental risk factors. However, the extent and order of event for each of these factors on disease contribution remains unclear.

Metal/metalloid imbalance has been implicated in various human diseases including cancer, cardiovascular disease, and neurodegenerative diseases. Correlation between heavy and trace metals such as lead (Pb), mercury (Hg), Chromium (Cr), arsenic (As), cadmium (Cd), aluminum (Al), manganese (Mn), magnesium (Mg), selenium (Se), nickel (Ni), copper (Cu), and zinc (Zn) have been investigated in a number of ALS studies (Al-Chalabi and Hardiman, 2013; Bocca et al., 2015; Dickerson et al., 2020; Farace et al., 2020). However due to varied methods, medium, populations studied, and heterogeneity of the disease, there exist large variations between studies making replication of results difficult. To date, no study has found causative links between metal/metalloid imbalance and ALS onset. For instance, onset of ALS can be clinically classified as either spinal, muscle weakness starting in the limbs, or bulbar, symptoms characterized by difficulties in swallowing and speech. Progression rate of bulbar onset ALS tend to be faster than spinal onset, and is considered the more severe variant. Males are more likely to develop spinal onset, while females have increased likelihood of developing bulbar onset (Logroscino et al., 2010). Men (1:350) also have higher lifetime risks than women (1:400) (Colombrita et al., 2009; Chang et al., 2012; van Es et al., 2017). Current prognosis for survival is 2–5 years after initial diagnosis (Miller et al., 2009; Brown and Al-Chalabi, 2017; Hulisz, 2018). Bulbar onset, lower ALS Functional Rating Scale (ALSFRS-R) score, and older patients often show lower survival rate (Kiernan et al., 2011). Additionally, ALS incidence differs by geographical region and ethnicity. Currently, most epidemiological studies have been based on European populations, which have an incidence rate of 2–3 cases per 100,000 individuals (Logroscino and Piccininni, 2019). Notably, regions with relatively more homogenous populations such as Scotland and Ireland, incidence rates are particularly high (2.6/100,000 individuals). On the contrary, East and South Asia have shown lower incidence rates (0.7–0.8/100,000 individuals), although there is still a lack of epidemiological studies for individual countries in these regions (Logroscino et al., 2010; Joensen, 2012; Chiò et al., 2013; Marin et al., 2014). In addition to incidence, survival rate also varies greatly by geography. Specifically, European populations have been evidenced to have shorter (24 months) survival time than Central Asian populations (48 months) (Marin et al., 2016). Representational studies from a diverse range of geographical regions are necessary for deeper understanding the roles of genetics and metal/metalloid imbalance on ALS disease pathogenesis.

In this study, we aim to assess potential differences in levels of heavy and trace metals/metalloids found in the cerebral spinal fluid (CSF) of ALS patients and corresponding controls. Specifically, we examined levels of copper (Cu), nickel (Ni), mercury (Hg), arsenic (As), manganese (Mn), and iron (Fe) using ICP-MS in 29 ALS patients and 9 controls from a cohort based in Shaanxi, China (Northwestern region). Findings from this study reveal onset- and sex-dependent metal dyshomeostasis in ALS patient CSF as well as potential mixture effects.

## Subjects and methods

### Study population

Twenty-nine sporadic cases diagnosed with definite ALS according to the revised El Escorial criteria and 9 age- and sex-matched controls recruited from the First Affiliated Hospital of Xi’an Jiaotong University (China) were included in this observational study. The control group consist individuals with non-neurodegenerative diseases such as headache and lower pack pain. As part of routine hospital visit, baseline demographic information, location of onset, ALSFRS-r score, smoking, drinking and exposure history, and laboratory test results were collected. All demographic and clinical information were collected by medical professionals. All participants provided informed consent prior to the procedures. This study was approved by the Institutional Ethical Committee of Xi’an Jiaotong University.

### Cerebral spinal fluid collection

CSF samples were obtained by lumbar pucture in the L3/L4 or L4/L5 interspace and collected into trace element free polypropylene tubes in 1 mL aliquots. All procedures were conducted at the First Affiliated Hospital of Xi’an Jiaotong University. Samples were gently mixed and centrifuged at 2,000*g* at 4°C for 10 min to eliminate insoluble materials and cells. Immediately after, samples were deep frozen and stored at −80°C until further analysis at Xi’an Jiaotong University iHarbor Research Center. Samples were thawed on ice prior to analysis.

### Metal/metalloid analysis

Aliquots of 200 μL CSF samples were diluted 10 folds with 65% Nitric Acid (Sinpharm Chemical Reagent Co., Ltd) and 31% hydrogen peroxide. Samples were dissolved on heat block (AS ONE, CHP-250DF, Japan) at 150°C. All metals [selenium (Se), copper (Cu), nickel (Ni), mercury (Hg), arsenic (As), manganese (Mn), cadmium (Cd), chromium (Cr), iron (Fe), and lead (Pb)] were quantified using Inductively Coupled Plasma-Mass Spectrometer (ICP-MS, PerkinElmer, NexION^®^ 350D, USA) in Nebulizer Gas Flow STD/KED (Instrumental parameters: Nebulizer Gas Flow 0.85 L/min, Auxiliary Gas Flow1.45 L/min, Mass range: 1∼260 a.m.u, Dark noise <0.2, Sensitivity: >105 cps/ppb 115 In, Long term stability: <4%, Precise of isotopic ratio: <0.1%). All blank samples were analyzed concurrently as the collected samples to ensure accuracy. The limit of quantification (LOQ) for each element were calculated and expressed as μg/L for all the metals 12.5 Selenium (Se), 0.475 Copper (Cu), 0.1 Nickel (Ni), 0.05 (Hg), 0.175 Arsenic (As), 0.55 Manganese (Mn), 0.01 (Cd), 4.8 (Cr), 21.475 (Fe), 1.225 (Pb). Because measurements for Se, Cd, Cr, and Pb were below the LOQ, results for these metal/metalloids have been omitted.

### Serum triglyceride quantification

Blood tests were performed at the First Affiliated Hospital of Xi’an Jiaotong University during patients’ first visit to the hospital (after experiencing first symptoms). Fresh blood samples (after overnight fasting) were tested for serum triglyceride.

### Statistical analysis

Statistical analyses were performed using the GraphPad Prism 8.0 software and R (ggplot2). All demographic information was presented as frequencies along with percentages. Comparative analyses of each metal/metalloid between groups were carried out using non-parametric pair-wise Mann–Whitney tests, *p*-value <0.05 was considered statistically significant. Results are presented as medians, 25th, and 75th interquartile ranges (IQR).

## Results

### Baseline demographic data

A total of 38 participants were included in this study, 29 ALS cases, and 9 controls. As shown in Table 1, 4 females (44.44%) and 5 males (55.56%) were included in the control group. For the ALS group, 16 were female (55.17%) and 13 were male (44.83%). The median age was 59.1 years (51–73) and 55.63 years (42–69) for the control and ALS groups, respectively. The average ALSFRS-R scores for the ALS patients was 41.73 (21–48). We further divided the ALS group into spinal (*n* = 20) and bulbar (*n* = 9) onset groups. In the spinal onset group, 9 cases were female (45%) and 11 were male (55%). In the bulbar onset group, there was a higher percentage of female cases (77.78%, *n* = 7) compared to male cases (22.22%, *n* = 2). The median ages for the spinal and bulbar onset groups were 55.7 (42–69) and 55.5 (44–67) years, respectively. The average ALSFRS-R score for the spinal group was 40.9 (21–47) and 43.4 (34–48) for the bulbar group.

**TABLE 1:** Baseline demographic characteristics for ALS cases and controls.

	Control		ALS	
	Non-ALS (*N* = 9)	Total (*N* = 29)	Spinal onset (*N* = 20)	Bulbar onset (*N* = 9)
**Sex**				
Females	4 (44.44%)	16 (55.17%)	9 (45%)	7 (77.78%)
Males	5 (55.56%)	13 (44.83%)	11 (55%)	2 (22.22%)
Median age (years)	59.1 (51–73)	55.63 (42–69)	55.7 (42–69)	55.5 (44–67)
Average ALSFRSR	–	41.73 (21–48)	40.9 (21–47)	43.4 (34–48)

### Cerebral spinal fluid metal/metalloid levels

**FIGURE 1:**
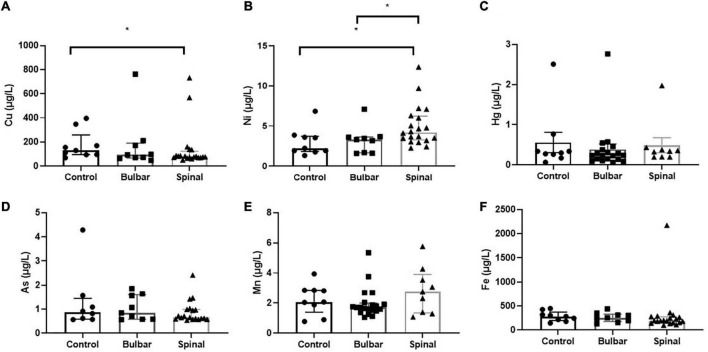
The scatterplots illustrate CSF Cu **(A)**, Ni **(B)**, Hg **(C)**, As **(D)**, Mn **(E)**, and Fe **(F)** levels found in control, bulbar-, and spinal-onset ALS patients. Data presented indicate median and IQR. *p < 0.05.

**FIGURE 2:**
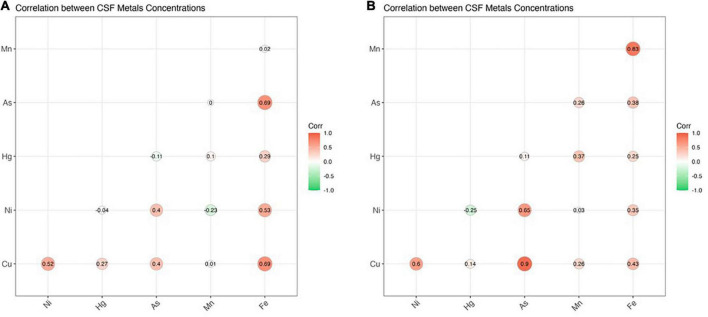
The figure illustrates correlation coefficients for CSF metal/metalloids in ALS cases **(A)** and healthy controls **(B)**. Significant correlations (*p* < 0.05) were found for the following metals: Cu/Ni, Cu/As, Ni/As, Fe/Cu, Fe/Ni, and Fe/As (ALS) as well as Cu/As (control). Thresholds of significance are as follows: 0.85 for healthy controls and for 0.3 for ALS cases.

ICP-MS was used to measure levels of Cu, Ni, Hg, As, Mn, and Fe in CSF of ALS patients and corresponding controls. Data were analyzed at multiple levels to thoroughly examine variation in metal/metalloid levels based on disease onset, progression, as well as sex and potential synergistic effects. First, we analyzed differences in metal levels between ALS patients and corresponding controls. Interestingly, contrary to previous findings, none of the metals in our study were found significantly different between control and ALS groups except for Cu, which was found lower in the ALS group (Cu = 80.12 μg/L, *p* = 0.05) compared to the control group (Cu = 129.7 μg/L) (Table 2). Noted, confounding factors including age, smoking, drinking, education level, and BMI showed no significant effects. Copper deficiency in ALS patients has also been reported in previous studies (Weihl and Lopate, 2006; Barros et al., 2018). Second, we divided the ALS group into bulbar and spinal onset groups to examine potential differences in metal levels between different types of ALS onset (Figure 1). As shown in Table 3, Cu levels were significantly lower in the spinal group (Cu = 78.11 μg/L, *p* = 0.04) compared to the control group (Cu = 129.7 μg/L). However, there was no noteworthy difference in Cu levels between bulbar onset and control groups. These results suggest that low Cu levels found in the ALS group may be predominantly contributed by spinal onset patients. In addition, Ni levels were significantly higher in the spinal group (Ni = 4.21 μg/L) compared to both control (Ni = 2.23 μg/L, *p* = 0.02) and bulbar (Ni = 3.30 μg/L, *p* = 0.03) groups. These results indicate potential differences in metal levels between bulbar and spinal onset patients, and that low Cu and high Ni levels may be associated with spinal onset. Third, we analyzed potential sex-dependent differences in CSF metal levels by dividing ALS cases into male and female groups. As shown in Table 4, there were no significant differences in CSF metal levels between female and male ALS patients. This suggests that sex may not be a confounding factor in CSF metal/metalloid levels for ALS patients. Fourth, to examine potential correlations between metal levels and ALS disease severity, we performed Pearson correlation analysis, and found that CSF metals were not correlated with disease severity (Table 5). Lastly, we conducted correlational assessment to examine potential associations between metals. As shown in Figure 2A, positive and significant correlations were found for the following metals: Cu/Ni, As/Cu, As/Ni, Fe/Cu, Fe/Ni, and Fe/As. To confirm whether these correlations are specific to ALS cases, we also conducted a correlation analyses for the control group. As shown in Figure 2B, significant correlations were found for As/Cu. This suggests correlations between As and Cu may not be specific to ALS patients. Correlations between Cu/Ni, As/Ni, Fe/Cu, Fe/Ni, and Fe/As in ALS patients may be interesting to investigate for future studies. These results are interesting as many of the metals listed such as Mn, Hg, and As were not found significantly different in the above analyses, although synergistic or mixture effects between metals have also been reported in other ALS studies (Figueroa-Romero et al., 2020).

**TABLE 2:** CSF metal/metalloid levels (median, 25th, and 75th percentile) for ALS cases and controls.

Metal/metalloid (μg/L)	Control *N* = 9 Median (IQR)	ALS *N* = 29 Median (IQR)	*P-value*
Cu*	129.70 (95.55–258.7)	80.12 (70.25–138.5)	0.05
Ni	2.23 (1.84–3.73)	3.66 (3.10–5.52)	0.07
Hg	0.27 (0.10–0.76)	0.26 (0.16–0.38)	0.61
As	0.90 (0.59–1.55)	0.70 (0.58–1.24)	0.52
Mn	2.07 (1.39–2.85)	1.75 (1.43–2.72)	0.51
Fe	283.0 (197.8–371.9)	201.3 (157.1–296.0)	0.19

**TABLE 3:** Distribution of CSF metal/metalloid levels (median, 25th, and 75th percentile) by disease onset.

Metal/metalloid (μg/L)	Control *N* = 9 Median (IQR)	Spinal onset *N* = 20 Median (IQR)	Bulbar onset *N* = 9 Median (IQR)
Cu*	129.7 (95.55–258.7)	78.11 (70.71–124.1)	93.37 (66.78–191.7)
Ni*	2.23 (1.84–3.73)	4.21 (3.27–6.23)	3.30 (1.65–3.64)
Hg	0.30 (0.22–0.55)	0.34 (0.20–0.41)	0.23 (0.14–0.38)
As	0.90 (0.59–1.55)	0.67 (0.59–1.01)	0.84 (0.57–1.59)
Mn	2.07 (1.39–2.85)	2.76 (1.34–3.91)	1.71 (1.48–2.01)
Fe	283.0 (197.8–371.9)	193.2 (151.5–274.3)	254.5 (177.6–330.2)

*Mann–Whitney test shows significant difference between Spinal and Control groups for Cu (*p* = 0.04) and Ni (*p* = 0.02), as well as between Bulbar and Spinal groups for Ni (*p* = 0.03).

**TABLE 4:** Distribution of CSF metal/metalloid levels (median, 25th, and 75th percentile) by sex for ALS cases.

Metal/metalloid (μg/L)	Female *N* = 16 Median (IQR)	Male *N* = 13 Median (IQR)	*P-value*
Cu	78.11 (67.27–169.2)	81.96 (70.25–90.40)	0.68
Ni	3.69 (3.04–6.52)	3.51 (3.10–5.52)	0.68
Hg	0.29 (0.09–0.44)	0.19 (0.09–0.32)	0.45
As	0.97 (0.64–1.52)	0.61 (0.57–0.70)	0.11
Mn	1.74 (0.54–2.88)	1.67 (0.99–3.62)	0.63
Fe	266.2 (161.7–311.1)	185.6 (157.1–246.9)	0.12

**TABLE 5:** Association between CSF metal/metalloid levels and ALSFRS-R scores.

Metal/metalloid	Correlation coefficients	*P-value*
Cu	0.05	0.77
Ni	–0.02	0.91
Hg	–0.13	0.56
As	0.04	0.83
Mn	0.30	0.20
Fe	0.03	0.87

### Correlation of cerebral spinal fluid metal/metalloids with serum triglyceride

**FIGURE 3:**
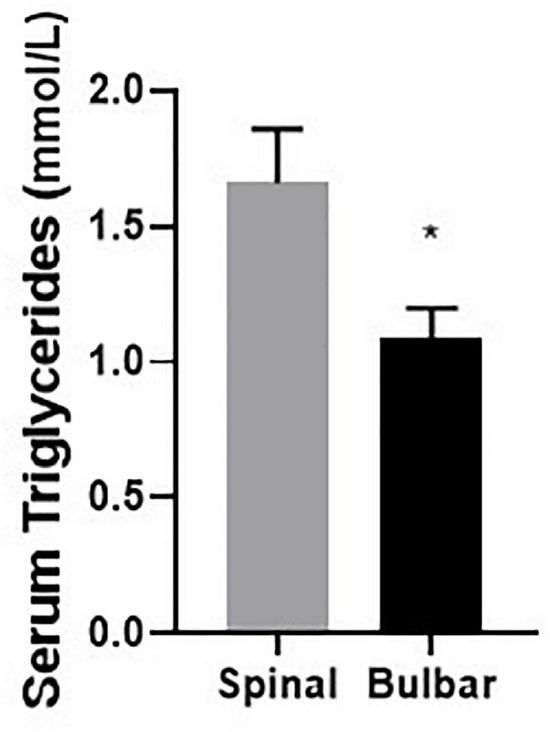
Serum triglyceride level is significantly higher in the spinal group compared to the bulbar group. Data presented for all ALS cases indicate mean and standard error. *p < 0.05.

Further analyses were conducted to examine potential correlation between CSF metal/metalloid levels with clinical features in ALS patients (Table 6). Routine laboratory test results included data on serum glucose, cholesterol, triglyceride, T3/T4 levels, etc. Interestingly, these clinical biomarkers were not found correlationed with CSF metal/metalloids levels. In addition, while triglyceride levels did not correlate with disease severity, the spinal group showed significantly higher levels than the bulbar group (Figure 3). In addition, we did not find significant correlation between ALSFR-S and triglyceride levels. Follow-up studies with additional triglyceride and ALSFRS-R data can be used to examine change in ALSFR-S versus change in triglyceride levels. This may reveal more about the potential relationship between tryglyceride and ALS disease progression. While serum triglyceride is mainly used as a biomarker for metabolic and cardiovascular disease, recent studies have indicated potential links to neurodegenerative diseases (Nägga et al., 2018; Bernath et al., 2020; Liu et al., 2020). Our findings suggest that while serum triglyceride did not correlate with any of the CSF metal/metalloids, it may be associated with the more severe form of ALS.

**TABLE 6:** Association between CSF metal/metalloid levels and serum triglycerides for ALS cases.

Metal/metalloid	Correlation coefficients	*P-value*
Cu	–0.03	0.84
Ni	–0.11	0.58
Hg	0.34	0.13
As	–0.11	0.55
Mn	0.01	0.95
Fe	–0.30	0.12

## Discussion

Main findings from this study suggest that CSF metal/metalloid levels not only differed between controls and ALS patients, but also between ALS patients with different forms of onset. Unlike some of the previous studies which have reported significant differences in various metals between control and ALS patients, our results indicate that only copper was significantly lower in the ALS group compared to the control group. Moreover, copper levels were especially low in spinal-onset patients. This result is in line with a previous study which showed lower CSF copper levels in spinal patients compared to bulbar patients (Patti et al., 2020). Copper imbalance has been reported in several neurodegenerative diseases including ALS, Alzheimer’s Disease, Menkes Disease, and Parkinson’s Disease (Telianidis et al., 2013; Chang and Hahn, 2017; Hilton et al., 2020; Qin et al., 2022). Currently, both high and low levels of copper have been correlated with ALS (Kapaki et al., 1997; Hozumi et al., 2011; Roos et al., 2013; Peters et al., 2016; Cicero et al., 2017; Qin et al., 2022). In ALS patient CSF samples, high copper levels have been reported by two research groups (Hozumi et al., 2011; Patti et al., 2020). On the other hand, a study led by Kapaki et al. demonstrated low copper levels in ALS patient CSF (Kapaki et al., 1997). Despite a lack of consensus between studies, which may be due to differences in participant characteristics (age, ethnicity, etc.), it seems that an imbalance in CSF copper levels may be correlated with ALS pathogenesis. Copper is an essential trace element that can cross the blood-brain barrier (BBB) via cerebral capillaries, which are mostly covered by astrocytes (Choi et al., 2009). In fact, concentrations of copper ions in the CNS (80 μM) are found higher than that of the blood (16 μM), muscle (10 μM), and lung (30 μM) (Hamilton et al., 1972). In the CNS, copper can be found in all parts of the brain and can function to promote neurotransmission, synaptic activities, free radical detoxification, and mitochondrial respiration (Gaier et al., 2013; Gil-Bea et al., 2017; Giampietro et al., 2018; Qin et al., 2022). Copper ions are incorporated into the cell via copper transporter 1 (CTR1) and divalent metal transporter 1 (DMT1) membrane proteins (Kuo et al., 2001; Lee et al., 2001; Arredondo et al., 2003). Once inside the cell, copper levels are closely regulated by efflux and influx pumps such as ATP7A (Tokuda and Furukawa, 2016). SOD1 is known to have high affinity for copper ions; changes in its expression level have been evidenced to influence copper levels in the spinal cord (Li et al., 2006; Lelie et al., 2011). However, copper imbalance has not been reported in SOD1-ALS cases, suggesting that ALS-related copper imbalance may be regulated via other trafficking systems. For example, various copper-requiring proteins and enzymes such as P-type ATPase, and cytochrome c oxidase receive copper ions via specific chaperones including HAH1, COX17, and CCS (Petris et al., 1996; Wong et al., 2000; Takahashi et al., 2002; Hamza et al., 2003). Notably, changes in ATP7A and CTR1 levels have also been found to affect copper accumulation inside the cell (Tokuda et al., 2013). In addition, mitochondrial copper has also been suggested to play a role in the pathophysiology of ALS, as mitochondrial dysfunction and metabolic defects represent important hallmarks for ALS motor neuron degeneration (Kong and Xu, 1998; Wiedemann et al., 2002; Muyderman and Chen, 2014; Carrì et al., 2017). Although no direct evidence of copper-induced mitochondrial dysfunction has been reported in the development of ALS, changes in certain copper-dependent enzymes such as PARK7, COX1, and COX2 have been shown to trigger neuronal death through regulation of mitochondrial function (Fujita et al., 1996; Borthwick et al., 1999; Wang et al., 2016). At the moment, there is no established consensus on whether high or low levels of copper is responsible for ALS, rather it seems that the imbalance of copper levels may be the key as both copper deficiency and accumulation can have compromising effects on normal cellular functions. Future studies cross-linking copper levels in the human body and potential changes in copper trafficking systems may provide further insight.

Next, while our findings indicate that there is no significant difference in CSF nickel levels between control and ALS groups. However, spinal-onset patients showed comparably higher nickel levels than both the control and bulbar groups. Nickel is considered an irritant, but also an essential and carcinogenic metal, known to accumulate in neuronal tissues, promote oxidative stress and mitochondrial damage, as well as inhibit neurotransmission (Saito et al., 2016; Song et al., 2017; Ijomone et al., 2018; Andrew et al., 2022). Previous reports have indicated that nickel is capable of stimulating the production of serum nitric oxide, which can interact with mitochondrial superoxide to form reactive peroxynitrite, thereby regulate neurotransmission (Cruz et al., 2004; Calabrese et al., 2010; Hattiwale et al., 2013). In a case-control study based on ALS patients in Denmark, Dickerson et al. found that women occupationally exposed to nickel had higher adjusted odds of developing ALS (Dickerson et al., 2020). In another study based on examining metal biomarkers in teeth, ALS cases were found to have 1.65 times more nickel than controls, indicating potential correlation between childhood metal uptake and later adulthood-onset (Figueroa-Romero et al., 2020). Interestingly, high nickel levels in body fluids of AD patients have been positively correlated with alcohol consumption and hepatotoxicity, and that nickel chelation may have beneficial effects on inhibiting Aβ42 peptide aggregation (Ormerod et al., 1997; Benoit and Maier, 2021). While nickel chelation may be a promising therapeutic strategy for ALS, potential difference in nickel levels between spinal and bulbar onset patients warrant further evaluation.

This study also examined potential sex-dependent differences in CSF metal/metalloid levels in ALS patients. We report that there was no significant differences in CSF metal/metalloid levels between male and female ALS patients nor healthy controls. A previous study based on participants from Bangladesh reported difference in selenium concentrations based on gender, and that males exhibited higher selenium levels than females. Depending on the dose, selenium can either be nutritional and toxic for the human body. Probable link has been reported between selenium toxicity and increased ALS risks (Vinceti et al., 1997). In particular, evidence suggests that selenium accumulation can induce neuronal apoptosis, and promote the translocation of copper/zinc SOD1 into the mitochondria (Xiao et al., 2006; He and Cui, 2021; Wandt et al., 2021). However, as with many metals, controversial results regarding selenium levels in ALS patients have also been reported. In particular, reduced blood selenium levels were found correlated with ALS in two other studies (Moriwaka et al., 1993; Peters et al., 2016).

In addition to onset- and sex-dependent analyses, we also examined potential associations between metals in ALS patients. In particular, correlations between Cu/Ni, As/Ni, Fe/Cu, Fe/Ni, and Fe/As were found specifically for ALS patients. Further investigation into the correlation between Fe and other CSF metal/metalloids may be interesting as it was found to be positively correlated with all tested metals except for Hg and Mn. Many of these metals such as mercury, arsenic, and manganese were not individually associated with either ALS onset or disease severity, although the results may be an indication of potential mixture effects between metals. Previous research has also indicated that exposure to metal mixtures may be positively correlated with disease outcome in ALS mouse model (Figueroa-Romero et al., 2020).

Over accumulation of triglycerides can be an important risk factor for cardiovascular disease. In the brain, imbalance of lipids or dyslipidemia can disrupt normal synaptic functions, membrane trafficking and protein activities (Blasco et al., 2017). Increased triglyceride level has been shown to be positively correlated AD risks (Nägga et al., 2018; Bernath et al., 2020). Yet, correlations between serum triglyceride levels and ALS risks have so far been inconsistent (Dorst et al., 2011; Blasco et al., 2017; Mariosa et al., 2017; Liu et al., 2020). High serum triglyceride levels were found correlated with prolonged life expectancy and better prognosis for ALS patients in several studies (Dorst et al., 2011; Nakamura et al., 2022). However, the difference in serum triglyceride levels between ALS patients and healthy controls are still controversial. One study found that serum triglyceride levels are found lower in ALS patients than in controls (Blasco et al., 2017). However, another study reported no significant difference in serum triglyceride between ALS patients and healthy controls (Chiò et al., 2009). To the best of our knowledge, this is the first study to examine potential associations between CSF metal/metalloids and serum triglyceride levels in ALS patients. Our results indicate that CSF metal/metalloids were not correlated with serum triglyceride levels in ALS patients. Previous epidemiology studies have linked heavy metal exposure to dyslipidemia. In particular, mercuy, lead, arsenic, copper, nickel, and cadmium were reported to be positively associated with serum triglyceride levels (Buhari et al., 2020; Ma et al., 2020; Kim et al., 2022). In animal studies, cadmium has been evidenced to enhance triglyceride accumulation through reduction of lipoprotein lipase activity (Barañski et al., 1983). Interestingly, serum triglyceride level did not correlate with ALS disease severity, although it was significantly higher in the spinal group compared to the bulbar group. Further confirmation is warranted, especially with the inclusion of a control group.

Results of this study is limited by the relatively small sample number and singularity in the type of sample used. Another major limitation of this study is that the measurements were only carried out once. We acknowledge that data interpretation can be limited by sample size and statistical imperfections. However, despite these limitations, this observational study presents valuable new information. Through the implementation of CSF samples, inclusion of stratified groups and analyses, we were able to gain insight into the potential association between CSF metal/metalloid levels with different forms of ALS onset, disease severity, sex, and serum triglycerides. Further validation is necessary with a larger participant pool and the incorporation of various sample types including urine, blood, and hair. In addition, it remains unclear whether these findings can be extended to other populations, although we provide new insights from a less studied geographical region. Lastly, future studies may also consider examining both genetic changes and metal exposure to gain further insight, including RNA-seq analyses to examine potential change in gene expression along with metal/metalloid levels.

## Conclusion

Metal homeostasis in the CNS is critically important for normal cellular processes and brain function. Our study examined potential associations of six CSF metal/metalloids with onset- and sex-specific ALS risks. Main findings identified that CSF copper and nickel levels differed by form of ALS onset. We conclude that while little difference was found between control and ALS groups, our results highlight the association of CSF metal/metalloids with ALS onset, sex, and disease severity.

## Data availability statement

The original contributions presented in this study are included in the article/supplementary material, further inquiries can be directed to the corresponding authors.

## Ethics statement

The studies involving human participants were reviewed and approved by Institutional Ethical Committee of Xi’an Jiaotong University. The patients/participants provided their written informed consent to participate in this study.

## Author contributions

QC and JD conceived the study. PW, TW, XQ, RJ, RZ, JJ, FH, and XX performed the material preparation, data collection, and analysis. QC, PW, and JD wrote the manuscript. All authors provided comments on draft versions, read, and approved the final manuscript.
